# Microbial diversity and community composition of caecal microbiota in commercial and indigenous Indian chickens determined using 16s rDNA amplicon sequencing

**DOI:** 10.1186/s40168-018-0501-9

**Published:** 2018-06-23

**Authors:** Ramesh J. Pandit, Ankit T. Hinsu, Namrata V. Patel, Prakash G. Koringa, Subhash J. Jakhesara, Jalpa R. Thakkar, Tejas M. Shah, Georgina Limon, Androniki Psifidi, Javier Guitian, David A. Hume, Fiona M. Tomley, Dharamshibhai N. Rank, M. Raman, K. G. Tirumurugaan, Damer P. Blake, Chaitanya G. Joshi

**Affiliations:** 1 Department of Animal Biotechnology, College of Veterinary Science and Animal Husbandry, Anand Agricultural University, Anand, Gujarat 388001 India; 2 Department of Animal Genetics and Breeding, College of Veterinary Science and Animal Husbandry, Anand Agricultural University, Anand, Gujarat 388001 India; 3 Department of Pathology and Population Sciences, Royal Veterinary College, North Mymms, Hertfordshire, UK; 4 The Roslin Institute, University of Edinburgh, Easter Bush, Midlothian, UK; 5 Department of Clinical Science and Services, Royal Veterinary College, North Mymms, Hertfordshire, UK; 6 Department of Veterinary Parasitology, Madras Veterinary College, Tamil Nadu Veterinary and Animal Sciences University, Chennai, 600007 India; 7 Translational Research Platform for Veterinary Biologicals, Tamil Nadu Veterinary and Animal Sciences University, Chennai, 600051 India

**Keywords:** Amplicon sequencing, Chickens, Microbiome, Pathogens, 16S rRNA gene

## Abstract

**Background:**

The caecal microbiota plays a key role in chicken health and performance, influencing digestion and absorption of nutrients, and contributing to defence against colonisation by invading pathogens. Measures of productivity and resistance to pathogen colonisation are directly influenced by chicken genotype, but host driven variation in microbiome structure is also likely to exert a considerable indirect influence.

**Methods:**

Here, we define the caecal microbiome of indigenous Indian Aseel and Kadaknath chicken breeds and compare them with the global commercial broiler Cobb400 and Ross 308 lines using 16S rDNA V3-V4 hypervariable amplicon sequencing.

**Results:**

Each caecal microbiome was dominated by the genera *Bacteroides*, unclassified bacteria, unclassified Clostridiales, *Clostridium*, *Alistipes*, *Faecalibacterium*, *Eubacterium* and *Blautia*. Geographic location (a measure recognised to include variation in environmental and climatic factors, but also likely to feature varied management practices) and chicken line/breed were both found to exert significant impacts (*p* < 0.05) on caecal microbiome composition. Linear discriminant analysis effect size (LEfSe) revealed 42 breed-specific biomarkers in the chicken lines reared under controlled conditions at two different locations.

**Conclusion:**

Chicken breed-specific variation in bacterial occurrence, correlation between genera and clustering of operational taxonomic units indicate scope for quantitative genetic analysis and the possibility of selective breeding of chickens for defined enteric microbiota.

**Electronic supplementary material:**

The online version of this article (10.1186/s40168-018-0501-9) contains supplementary material, which is available to authorized users.

## Background

Ensuring the secure supply of safe food is a major global concern. Increasing human population sizes, income levels and urbanisation all contribute to rising demand for protein, and therefore livestock [1, 2]. However, key constraints on animal production include infectious diseases, which can be exacerbated by suboptimal commensal microflora that undermine capacity to thrive [3].

Chickens are the most numerous livestock in the world, with relatively low production costs and highly efficient food conversion. More than 60 billion chickens are produced annually, with production predicted to increase further over the next 20 years, particularly in South Asia and Africa [4]. The importance of the caecal microbiome to chicken health and productivity has long been recognised, especially in food conversion, resistance to disease and colonisation by zoonotic pathogens [5–8]. In line with other next-generation sequencing (NGS) microbiome studies [9–15], we have previously identified *Bacteroidetes*, *Firmicutes* and *Actinobacteria* as highly abundant phyla within chicken caecal populations, while functional metagenomics analysis revealed enrichment of sequences corresponding to carbohydrate metabolism [16]. However, the influence of host genetics on caecal microbiome structure is unclear [17–19].

Reports of higher productivity, product quality and/or pathogen resistance have been attributed to some indigenous chicken breeds. For example, 17 breeds of Indian origin are currently registered with the National Bureau of Animal Genetic Resources (NBAGR) at the Indian Council of Agricultural Research (ICAR) (http://www.nbagr.res.in/regchi.html). Among these breeds examples such as Aseel and Kadaknath have been associated with improved egg, meat and welfare traits such as reduced feather pecking [20, 21], resistance to infectious disease [22, 23] and immune parameters distinct from those of modern commercial chickens [24, 25]. Variation in immunity-related pathways can influence microbiome structure in humans [26] and is also likely to affect chicken microbiomes [27]. Analysis defining the caecal microbiome of native Indian chicken breeds of Assam state has been reported recently [7], although the significant impact of environmental factors makes it impossible to compare between microbiome studies [28]. Evidence from humans, mice and chickens suggest that host genotype can exert a strong influence on microbiome composition [18, 19] and thereby offers a quantifiable phenotype which may differ between chicken breeds and individuals, and may be amenable to genetic selection. Here, we use NGS targeting hypervariable regions within microbial 16S rRNA genes to compare the caecal lumen microbiota of commercial broiler-type lines, which are comparable to those used around the world, to indigenous Indian breeds reared in parallel under controlled commercial-type management conditions. Our hypothesis was that the caecal lumen microflora would vary significantly between chicken breeds and lines, offering opportunities for targeted genetic improvement by selective breeding. Given the importance of primer choice to successful NGS [29–34], we also compared multiple primers targeting different variable regions of the 16S rRNA.

## Methods

### Chicken breeds and experimental design

Four chicken breeds or lines were chosen for comparison in this study including two indigenous Indian breeds and two global commercial broiler lines. The indigenous breeds Kadaknath and Aseel were chosen for use at the Central Poultry Research Station of Anand Agricultural University, (AAU, Gujarat, India; termed location 1) and duplicated at Tamil Nadu Veterinary and Animal Sciences University (TANUVAS, Chennai, Tamil Nadu, India; location 2) (Additional file 1). Commercial Broilers of the Cobb400 (AAU) and Ross 308 (TANUVAS) lines were used for comparison as representatives of the local dominant-intensive commercial production systems [35].

At each location, ten chickens of each breed were hatched on the same day and reared in neighbouring pens in single poultry houses, providing independent replication and controlling against local spatial variation. Chickens were accommodated in this manner to avoid husbandry (behavioural) problems from arising when the chicken breeds were mixed. Care was taken to ensure that pens were of equal sizes, equidistant to the door and light sources and received exactly the same husbandry to minimise non-host variation. A deep litter system was employed using rice husk as a substrate in common with local practices. All chickens were fed a standard maize and soybean-based commercial diet which included bacitracin methylene disalicylate (BMD) and maduramycin (10%) for routine prophylaxis.

### Sample collection and DNA extractions

At each location, five apparently healthy chickens were selected at random from each group, caught and euthanized by cervical dislocation at 42 days of age. Both caecal pouches were opened immediately using sterile scissors and the contents were recovered into sterile cryovials containing Bacterial Protect RNA reagent (QIAGEN, Germany) at an approximate 1:1 ratio (*w*/*v*). Each sample was immediately stored in a portable freezer at − 20 °C, transported to the laboratory and stored at − 80 °C.

Total genomic DNA was extracted from the pooled caecal contents of each individual chicken using the commercially available QIAamp Fast DNA Stool Mini kit (QIAGEN, Germany) following the manufacturer’s instructions with some modifications. Briefly, 300 μL of caecal content with Bacterial Protect RNA reagent was added to 1 mL of InhibitEX buffer, vortexed at 2800 rpm for 1 min to homogenise and incubated at 80 °C for 10 min. The mixture was then centrifuged at 2600 g for 30 s to remove residual solid material and 600 μL of supernatant was processed as recommended by the manufacturer. DNA was treated with DNase free RNase (Macherey-Nagel, Germany) to remove contaminating RNA. DNA concentration and quality were assessed using a Qubit 2.0 fluorometer (Invitrogen, ThermoFisher scientific, MA) and gel electrophoresis. DNA was stored at − 20 °C until further processing.

### 16S rRNA gene amplification and MiSeq sequencing

Six hypervariable regions within the 16S rRNA gene were amplified from caecal DNA sample using three different primer pairs (Additional file 2). Each 25 μL PCR reaction comprised of 2.5 μL DNA (~5 ng/μL), 5 μL each forward and reverse primer (1 pM) and 12.5 μL 2X KAPA HiFi HotStart ReadyMix (Kapa Biosystems, UK). PCR amplification cycles were as follows, initial denaturation at 94 °C for 3 min, followed by 25 cycles of 98 °C for 20 s, X °C for 10 s and 72 °C for 12 s, where X was the annealing temperature optimised at 65, 60 and 66 °C for primer pairs 1, 2 and 3, respectively, and a final extension at 72 °C for 40 s. Amplicons were further processed for library preparation using Illumina’s Nextera XT library preparation kit (Illumina, USA). Sequencing was performed using an Illumina MiSeq desktop sequencer at the Department of Agricultural Biotechnology (a center of excellence in biotechnology, AAU, Anand). Trimming of adaptor sequences was performed using Illumina analysis software V2.5 as recommended by the manufacturer using default parameters.

### Sequence data analysis

All 16S rDNA reads were uploaded to the MG**-**RAST V3.6 open source online server for phylogenetic and functional classification of metagenomics data [36]. Low-quality reads were trimmed using SolexaQA [37] with default parameters in MG-RAST. Annotations were made against the RDP (Ribosomal Database Project) database with a minimum *e* value of 1E-5 and minimum identity of 80%. The data were further analysed using Statistical Analysis of Metagenomic Profiles (STAMP v2.1.3) [38], METAGENassist [39] and PAST v2.17c [40]. Comparative analysis for taxa in terms of percentage mean relative frequency was performed using STAMP, where Benjamini-Hochberg FDR was used for multiple test corrections to minimise false discovery rates during multiple group comparative analysis. Further, each taxonomic profile from STAMP was uploaded to METAGENassist for analysis where data were filtered for unassigned bacteria and samples were scaled to each other through normalisation using Pareto Scaling (mean-centered and divided by the square root of standard deviation of each variable) [41]. The processed data were subsequently used for correlation analysis. Principal coordinate analysis (PCoA), one-way ANOSIM and clustering were done using PAST. The processed quality filtered sequences were downloaded from the MG-RAST server and used to calculate diversity indices, 2D-PCoA (unweighted UniFrac) and rarefaction curve using QIIME where sequences were clustered at 97% similarity. Moreover, for rarefaction curve sequences were rarefied at 10,000 sequences per sample. Boxplots were generated using BoxPlotR [42]. One-way ANOVA was used to compare three primer pairs while Mann-Whitney test was used to compare differences among two locations within PAST software. To determine the core microbiome of chicken breeds under study, genus abundance > 0.1 and > 1.0% were taken into account and Venn diagrams were generated using Venny 2.1 [43]. To identify chicken line-specific biomarkers at multiple taxonomical levels, the bacterial abundance profile of birds pooled by (i) breed/line and (ii) location were analysed using linear discriminant analysis effect size (LEfSe) [44, 45]. For this analysis, bacterial abundance profiles were calculated at taxonomic levels from phylum to genus in %. For LEfSe analysis, the Kruskal-Wallis test (alpha value of 0.05) and LDA score of > 3.5 were used as thresholds.

## Results

### Microbiome sequencing

Caecal contents were collected from 30 chickens, split equally between locations 1 and 2 at the Anand Agricultural University (AAU, Gujarat, India) Central Poultry Research Station, and Tamil Nadu Veterinary and Animal Sciences University (TANUVAS, Chennai, Tamil Nadu, India), respectively. Fifteen birds sampled from location 1 included five from each of the indigenous Indian Aseel and Kadaknath breeds, supplemented by five commercial Cobb400 broilers for comparison. Fifteen birds sampled at location 2 duplicated those sampled from location 1, with the exception that Ross 308 commercial broilers were used instead of Cobb400, representing the local dominant-intensive commercial production systems [35]. All chickens were sampled at 42 days of age. Total DNA was isolated and amplified using three sets of primers targeting the 16S rDNA hypervariable regions V1–V2, V3–V4 and V5–V6. We generated 10.35 million sequences corresponding to 19.41Gbp of data in 90 files (Additional file 3). The average number of sequences per sample was 115,010 (Table 1).

**Table 1:** Summary statistics of sequences analysed including average OTU numbers detected and microbial diversity covered. For each chicken breed or line, the sequencing reads of all three primer pairs were merged and OTUs were clustered at > 97% similarity using QIIME

Location	Breed/line	Total sequences	Av. sequences/ sample	Av. no. OTUs	Average microbial diversity covered (% Good’s coverage)
1	Aseel	839,375	55,958	1134	94.6
	Kadaknath	1,042,538	69,503	816	95.9
	Cobb400	679,158	45,277	1273	94.1
2	Aseel	2,775,511	185,034	735	95.7
	Kadaknath	2,548,471	164,390	833	95.6
	Ross 308	2,465,845	169,898	645	97.0

### Operational taxonomic unit (OTU) occurrence and data rarefaction

**Fig. 1:**
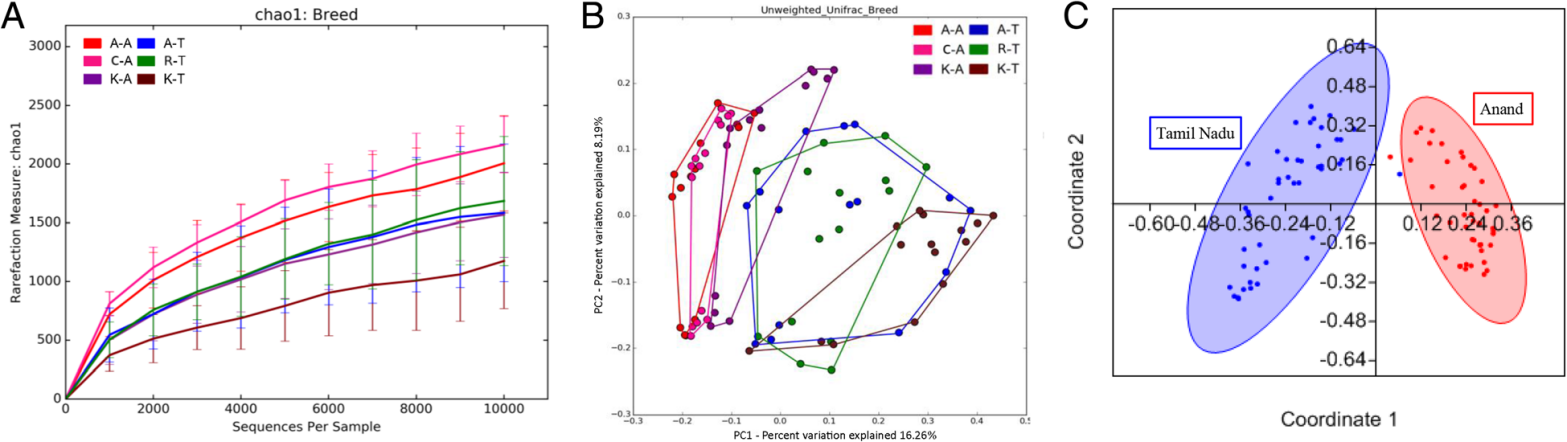
Rarefaction curve and PCoA for each breed and location sampled, created by combining data from all three primer pairs. **a** Rarefaction and **b** PCoA for each individual chicken breed or line was generated using QIIME where sequences were clustered at 97% similarity and rarified at > 10,000 sequences per sample. **c** PCoA based on location was generated using the Bray-Curtis distance method using PAST. A-, C-, R- and K- represent the Aseel, Cobb400, Ross 308 and Kadaknath chicken breeds or lines respectively. -A sampled at location 1 in Anand, -T sampled at location 2 in Tamil Nadu

Initially, caecal microbiome populations associated with each chicken breed or line were analysed based on different chicken lines irrespective of primer pairs. At both locations, the commercial broiler groups presented the highest average number of OTUs (Table 1). The indigenous Kadaknath breed presented the lowest number of OTUs in each duplicate study. Rarefaction based on the Chao1 index approached asymptotic for each breed or line, suggesting the availability of sufficient reads to represent each microbiome community (Fig. 1a), confirmed using the Shannon index and number of observed OTUs (Additional file 4). PCoA of the OTU data for each chicken breed or line revealed distinct, but overlapping, profiles including a high level of variation between geographical locations, supplemented by less pronounced but nonetheless distinct variation between breeds (Fig. 1b, c). Clustering analysis using 100 bootstraps in PAST also showed clear separation between the two locations (Additional file 5). Based on Good’s coverage index, on average 95.48% of caecal microbial diversity was covered in this study (Table 1).

### Comparison of primer performance

**Fig. 2:**
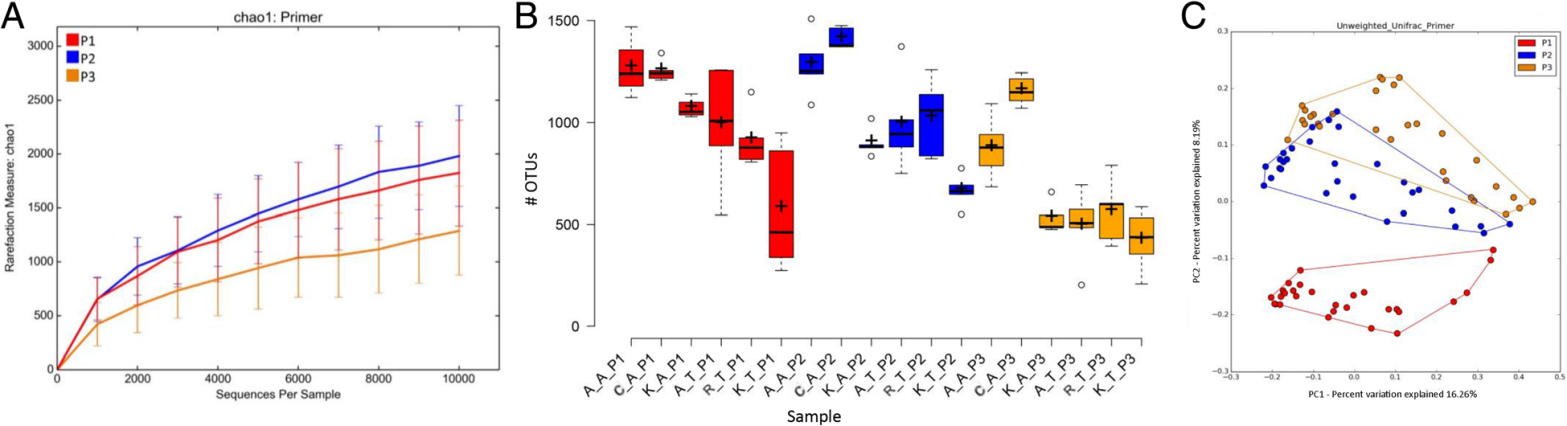
A rarefaction curve (**a**), box plot (**b**) and PCoA (**c**) for each primer pair. For analysis, respective sequences of each primer pair were clustered at 97% similarity using QIIME. For rarefaction plots, sequences were rarefied with 10,000 sequences per sample and the Chao1 index was plotted. PCoA was generated using unweighted unifrac metrics. Box plots were generated using BoxplotR

We then compared the three individual primer pairs to determine which pair provided the most detailed and discriminating profiles of the caecal microbiome for our samples. Comparative analysis revealed that species richness reflected in terms of rarefaction using the Chao1 index, calculated using 10,000 sequences subsampled from each sample (rarified), were highest for P2 compared to P1 and P3 (*p* < 9.6E-06) (Fig. 2a). The numbers of OTUs identified by each primer set were also highest using P2, and lowest using P3 (*p* < 1.8E-05) (Fig. 2b). Rarefaction based on the observed OTUs is shown in Additional file 6. On an average, 1000, 1040 and 674 OTUs were identified using primer pairs P1, P2 and P3, respectively. In addition to Chao1 and the number of observed OTUs, the values of other alpha diversity indices such as the abundance-based coverage estimator (ACE) (*p* < 4.2E-06) and phylogeny-based diversity estimator (PD whole tree) (*p* < 2.8E-06) were also highest for P2 and lowest for P3 (Additional file 7), although values from the Shannon and Simpson indices were less distinct. All diversity indices showed significant variation among the three primer-pairs (Additional file 7). Further, PCoA analysis revealed that P1 formed an isolated cluster, while P2 and P3 formed clusters with a partial overlap (Fig. 2c). Taxonomically, primer pair P3 revealed a far greater proportion of sequences attributed to the phylum *Bacteroidetes* (Additional file 8). MG-RAST taxonomy identifiers for sequences generated with each primer set for each chicken breed or line indicated an average of 537, 518 and 422 species from primer pairs P1, P2 and P3, respectively (Additional file 9). Primer pair P2, which spans the V3-V4 hypervariable regions of the 16S rRNA gene, produced the most detailed and discriminatory results and therefore the sequences generated using this primer set were used for all subsequent analysis of caecal microbiota.

### Variation in caecal microbiome structure between chicken breeds and lines

**Fig. 3:**
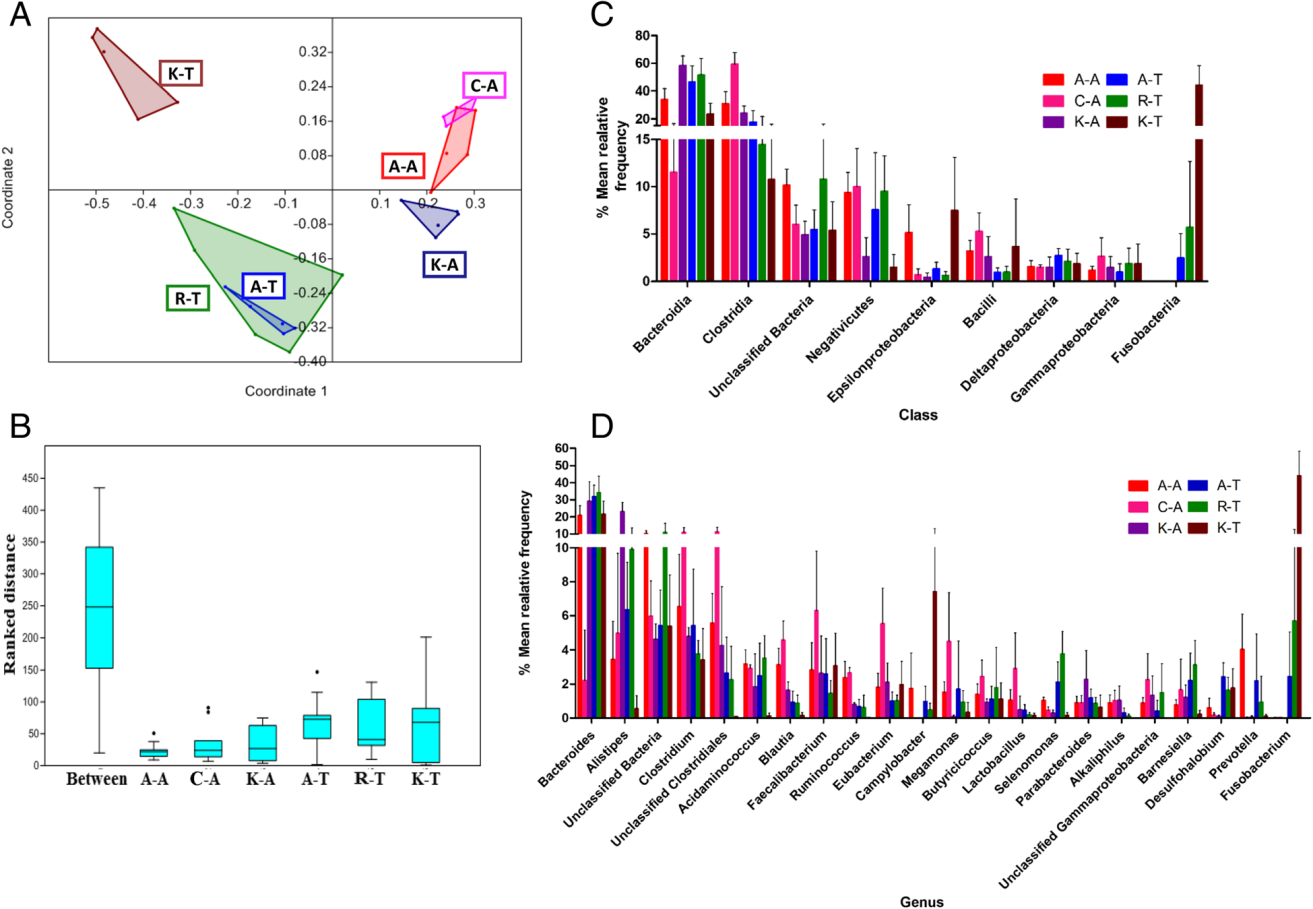
PCoA and class and genus level classification of caecal microbiomes from chicken breeds and lines reared at locations 1 and 2 (Anand and Tamil Nadu). Only sequencing reads produced using primer P2 were used for this analysis. **a** PCoA using the Bray-Curtis method in PAST. **b** Box plot indicating differences in the ranked distances in each group (see Additional file 10 for the pairwise comparison of *P* values by ANOSIM). **c**, **d** abundance of bacteria in the caeca of chicken lines at class and genus level respectively. Only classes and genera with abundance > 1.0% in any of the chicken lines was plotted. A-, C-, R- and K- represent the Aseel, Cobb400, Ross 308 and Kadaknath chicken breeds/lines respectively. -A sampled at location 1 in Anand, -T sampled at location 2 in Tamil Nadu

Figure 3a shows PCoA of the variation between microbiome profiles based upon Bray-Curtis dissimilarity. Coordinate 1, representing 30.1% of the variation, was associated with the different locations. Coordinate 2 (18.9% of the observed variation) revealed overlap between the Aseel and commercial broiler clusters within their respective experimental locations, while both were distinct from the Kadaknath clusters. ANOSIM analysis (*R* = 0.9097, *p* < 0.01, Sequential Bonferroni correction) also highlighted significant differences between chicken breeds (Fig. 3b, Additional file 10). Bacteroidetes was the dominant phylum in all chicken groups except Cobb400 and Aseel at location 1, where *Firmicutes* were more common (Additional file 11). Combined, these two phyla accounted for 76.6 to 90.8% of total bacterial sequences from the caeca of all chicken breeds or lines with the exception of Kadaknath birds at location 2 (46.2%), where Fusobacteria accounted for 41.3%. Comparing locations, Fusobacteria were more common in all chicken breeds or lines raised at location 2, indicating one or more environment-specific variable(s). Actinobacteria, together with bacteria left unclassified, were represented by between 8.5 and 18.3% of sequences from all chicken breeds or lines.

At class level classification, *Bacteroidia* (*p* < 8.9E-06) were predominant in all chicken breeds or lines except the Cobb400 reared at location 1, where *Clostridia* (*p* < 4.1E-07) were more common (Fig. 3c, Additional file 11). *Clostridia* were abundant in all chickens raised at location 1. Combined, the *Bacteroidia* and *Clostridia* represented the majority of classes within the caecal microbiota. Sequences representative of the class *Fusobacteriia* (*p* < 1.4E-07) were common in chickens sampled at location 2, explaining the high occurrence of the phyla *Fusobacteria* described above.

At genus level, *Bacteroides* (*p* < 7.2E-05) was found to be most common in all chicken breeds or lines except the commercial Cobb400 from location 1, where *Clostridium* (*p* < 2.1E-03) and unclassified *Clostridiales* (*p* < 1.3E-05) presented higher proportions. *Alistipes* (*p* < 1.0E-07) were also common in Kadaknath chickens at location 1. The other genera present, and their relative levels are indicated in Fig. 3d (see also Additional file 11).

Numbers of OTUs were high with both of the commercial broiler lines presenting 1411 and 1023 OTUs, followed by Aseel with 1285 and 992 OTUs and Kadaknath with 900 and 665 OTUs (locations 1 and 2, respectively). Comparing both locations, chickens raised at location 1 presented more OTUs than those reared at location 2 (*p* < 0.0025, Mann-Whitney test; Additional file 12).

### The core caecal microbiome, breed-specific biomarkers and correlations among bacteria

We pooled sequencing reads produced using primer pair P2 from each chicken breed or line into a single pool, combining samples from both locations. In total, 35 genera each represented more than 0.1% abundance in the core caecal microbiome of all breeds or lines (Additional files 13 and 14). Twenty one of the 35 genera belonged to the phylum *Firmicutes*, representing 11 different families. Seven and five genera belonged to the Bacteroidetes and Proteobacteria, respectively with the rest belonging to other diverse phyla. Total of 12 and 10 genera were uniquely detected in Aseel and Cob400 chickens s in excess of this threshold. Comparison of the datasets found *Geobacillus*, *Cyclobacterium*, *Caldicellulosiruptor*, *Thermobaculum*, *Caulobacte*, *Desulfovibrior* and *Cytophaga* in all Kadaknath birds, but not in any other dataset above the 0.1% threshold, and *Slackia*, *Cronobacter*, *Phascolarctobacterium*, Unclassified Alphaproteobacteria, *Oceanimonas*, *Deferribacter*, *Tepidimicrobium*, Candidatus Phytoplasma, *Atopobium*, *Tannerella*, *Zunongwangia* and *Acetobacterium* were all found to be specific to Aseel at the same threshold. Two and nine of these genera were identified in both locations for their respective breeds, indicating consistent breed-specific high-level colonisation. Twelve genera were found to exceed the 0.1% threshold in a single commercial broiler line (Cobb400: ten, Ross 308: two) but not an indigenous breed. Just ten genera were detected within the core (conserved) caecal microbiome between all breeds or lines studied when the threshold was increased to 1% abundance (Additional files 13 and 14).

**Fig. 4:**
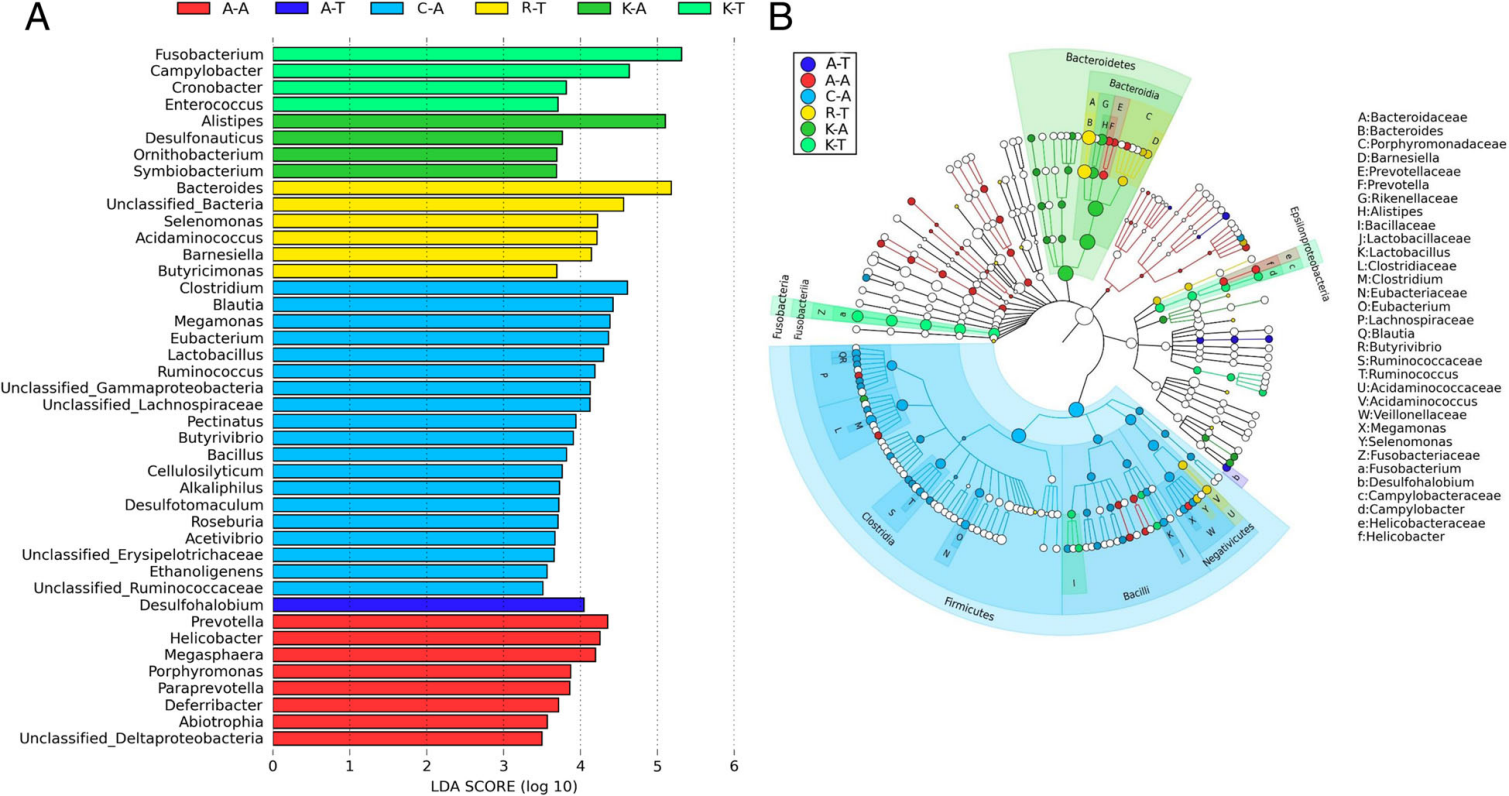
Chicken breed and line-specific biomarkers. **a** LEfSe analysis shows differentially abundant genera as biomarkers determined using Kruskal-Wallis test (*P* < 0.05) with LDA score > 3.5. **b** Cladogram representation of the differentially abundant families and genera (only top 50% are plotted here). The root of the cladogram denotes the domain bacteria. The taxonomic levels of phylum and class are labelled, while family and genus are abbreviated, with the colours indicating the breed/line hosting the greatest abundance. The size of each node represents their relative abundance

LEfSe analysis was performed with the pooled data to identify specific taxa that varied in abundance consistently by chicken breed or line across the locations and thus could be used as biomarkers. In total, 42 genera were identified with LDA scores > 3.5 (Fig. 4a). A cladogram for family and genus level abundance is shown in Fig. 4b. Comparison between locations 1 and 2 identified 17 genera with LDA scores > 3.5 (Additional file 15). If biomarkers which appeared at locations were excluded, 11 breed-specific biomarkers were present in Cobb400, 5 in Ross 308 and 4 in Aseel reared at locations 2 and 1, respectively. Four different biomarkers were present in the Kadaknath at each location, while Aseel reared at location 2 presented 1. The genera *Fusobacterium*, *Campylobacter*, *Cronobacter* and *Enterococcus*, which are known to include potential pathogens of poultry and/or humans, were biomarkers in the Kadaknath raised at location 2, with *Helicobacter* a biomarker at the same location in Aseel.

**Fig. 5:**
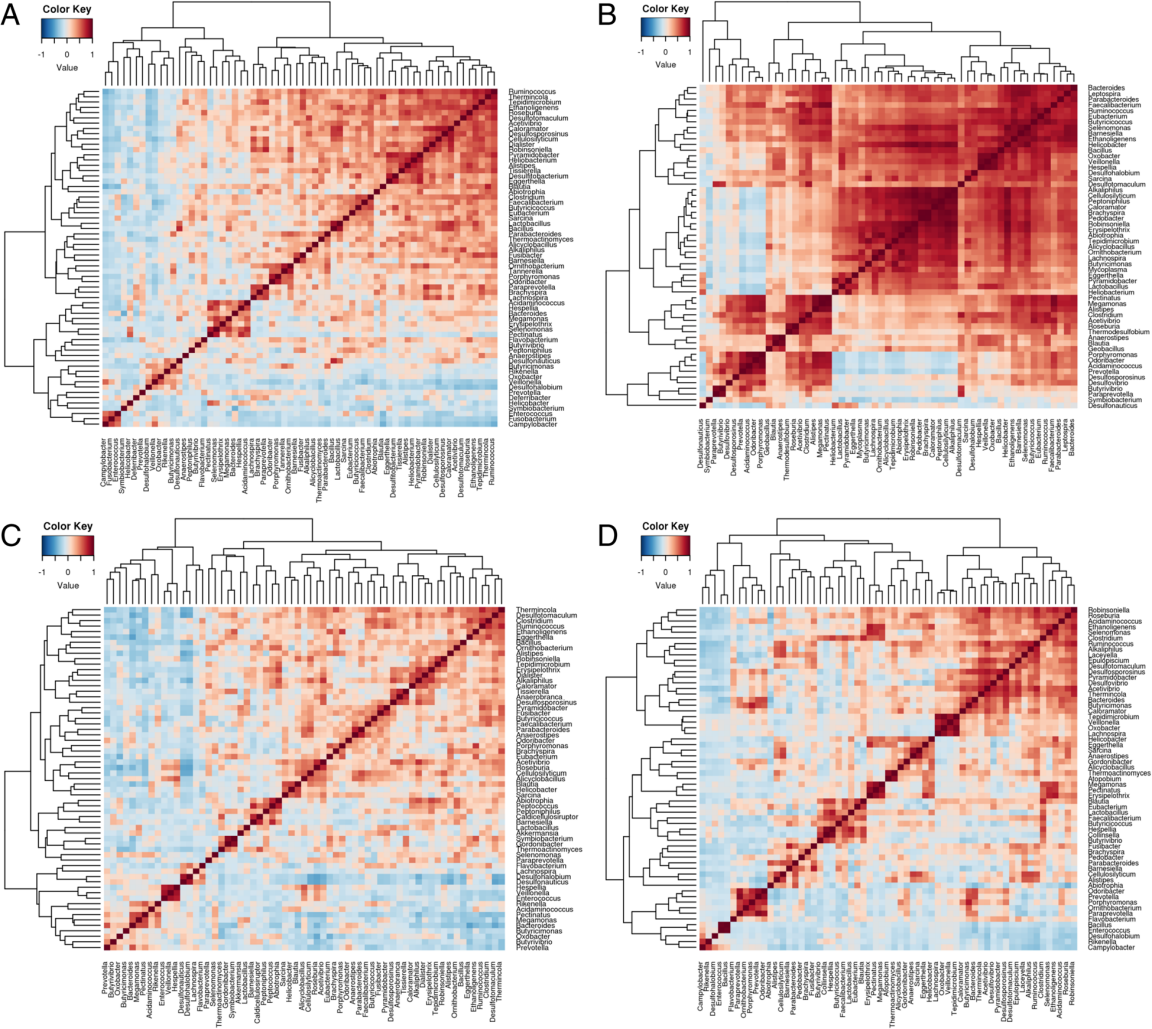
Correlation among the bacterial genera detected in the caeca of different chicken breeds. Sequencing reads produced using primer pair P2 were pooled into a single pool for each breed, combining samples from different farm locations. A Pearson’s *r* correlation was expressed using METAGENassist. The breeds represented are **a** Aseel, **b** Cobb400, **c** Ross 308 and **d** Kadaknath

For correlation analysis, data generated using primer P2 was pooled for each breed or line, and relative proportions at order and genus levels were produced using METAGENassist, expressed in terms of Pearson’s r correlation. Genera and order level correlations among microbes in each breed or line are shown in Fig. 5 and Additional file 16, respectively. For Aseel, the occurrence of genera which include potentially pathogenic species such as *Campylobacter*, *Fusobacterium*, *Enterococcus* and *Helicobacter* exhibited a positive correlation with each other. Surprisingly, *Prevotella*, *Rikenella* and *Butyricimonas* also showed negative correlations with most of the genera detected in Aseel (Fig. 5a). The majority of orders and genera detected in Cobb400 chickens were positively correlated, with few negative correlations (Fig. 5b and Additional file 16). Most genera were positively correlated within the Ross 308 caecal microbiome, although examples such as *Prevotella*, *Butyricimonas*, *Enterococcus* and *Bacteroides* were again negatively correlated (Fig. 5c.) In the Kadaknath genus level caecal microbiome, two clusters were detected with separate positive correlations. However, *Campylobacter*, *Rikenella*, *Enterococcus*, *Bacillus* and a few other genera showed negative correlations with most other genera (Fig. 5d). Cumulatively, Clostridiales, Bacteroidales, Spirochaetales, Synergistales and Flavobacteriales were positively correlated with each other in all chicken breeds or lines except Flavobacteriales in the Kadaknath. Variation in correlation was also observed between the two commercial broiler lines, for example in Cobb400, most bacteria were positively correlated with each other, while in Ross 308 *Butyrivibrio*, *Bacteroides*, *Megamonas*, *Desulphonauticus*, *Pectinatus*, in Aseel, *Fusobacterium*, *Helicobacter*, *Veillonella* and *Symbiobacterium*, and in Kadaknath *Rikenella* and *Bacillus* were correlated, with two genera *Enterococcus* and *Desulphohalobium* commonly presenting negative correlations with most others.

## Discussion

Demand for poultry meat and eggs is increasing dramatically, most notably in South Asia, where a mix of global commercial-type lines and indigenous chicken breeds are kept [35]. As efforts are made to improve the genetic merit of chickens, it is important to identify those genotypes best suited to the varied climatic regions and prevailing production systems, including those which favour a beneficial gut microbiota. The definition of a ‘beneficial’ microbiota is challenging, but comparison of microbiome composition and structure between chicken breeds that have been associated with improved production, health or other welfare traits can provide a valuable source of phenotypic data, with scope to inform genomic analyses to identify host sequence variation associated with microbiome composition. In addition, transplant of ‘beneficial’ microbiota might provide another strategy to improve both production and health in poultry. In inbred mice, transfer of microbiota between mice differing in susceptibility to the enteric pathogen *Citrobacter rodentium* resulted in a reciprocal transfer of susceptibility and resistance [46]. Here, we selected a single time point late in the broiler production cycle to permit assessment of the outcome of colonisation throughout each chickens’ life. It is important to note that enteric microbial populations will change over time as a consequence of variables such as the development of immune competence and environmental interactions. Future studies will explore fluctuations in microbial diversity as each chicken breed matures.

Here, we employed 16S rRNA amplicon sequencing as a measure of bacterial occurrence within chicken caecal lumen microbiota. We decided to focus on the lumen microbiota in order to standardise sample collection between chickens, and to permit comparison with previous studies by others [e.g. 15]; but recognise that that the mucosal-associated microbiota are likely to present different profiles. 16S rRNA amplicon sequencing has been used widely for classification of diverse microbial communities; however, the bacterial 16S rRNA gene includes nine hypervariable regions and sequences generated using different combinations of these regions commonly present varied profiles of microbial diversity. The optimal choice of hypervariable region(s) and primer combination vary between different ecological populations [47]. Here, we tested three primer sets spanning the V1–V3, V3–V4 or V5–V6 regions of 16S rRNA gene. Rarefaction curves based on the Chao1 index and the number of OTUs detected were comparably close to asymptotic between primer pairs, indicating that the sequences generated per sample were adequate to define and compare the bacterial diversity present within the samples. Further, the mean of Good’s coverage (an alpha diversity index) for all the samples was high (95%). The bacterial diversity, defined as the number of OTUs and species detected, was highest using primer set P2 (V3-V4) and least using P3 (V5-V6). Primer set P2 also illustrated greater species richness using the Chao1 index (accounting for rare OTUs), ACE index (an abundance-based coverage estimator) and PD whole trees (phylogeny-based diversity estimator). Primer set P3 generated a dataset apparently richer in *Bacteriodetes* sequences with lower total diversity. Based on these observations, we selected primers targeting the V3-V4 region, in agreement with the findings of others where primers targeting the V3, V4, V1–V3, V4–V6 hypervariable regions were compared [18].

The caecal lumen microbiomes of Indian chicken breeds evaluated here were dominated by sequences representative of the phyla *Firmicutes* and *Bacteroidetes.* Combined, these phyla commonly accounted for more than 80% of the total microbial populations detected. Earlier studies have highlighted similar proportions, with *Firmicutes* commonly dominant [7, 12, 13, 48]. Geographical location exerted a substantial impact on the variation between caecal microbiome populations, including variation in the *Firmicutes*/*Bacteroidetes* ratio. In the context of this study, the geographic separation of locations 1 and 2 included husbandry, dietary, climatic and other environmental variables. Such variables have been widely recognised to impact on microbiome structure and diversity [28]. Higher *Firmicutes*/*Bacteroidetes* ratios have been associated with human obesity [49, 50], and the reverse has been linked with weight loss [51]. Both *Firmicutes* and *Bacteriodetes* have been associated with short chain fatty acid metabolism, although more specifically *Firmicutes* contribute to butyrate and propionate synthesis, whereas *Bacteroidetes* primarily synthesise propionate. *Bacteriodetes* but not *Firmicutes* produce α-amylase, α-1,2-mannosidase and endo-1,4-β-mannosidase [52] and are more likely to break down starch and other polymeric substances. Another example of geographic variation was the occurrence of *Fusobacteria* and *Campylobacter*, which formed a major component of all chicken breeds raised at location 2 and appeared as a biomarker for the Kadaknath breed at that particular location. Class-level analysis found *Clostridia*, Gram-positive rod-shaped bacteria including the genera *Clostridium*, *Blautia*, *Butyrivibrio*, *Ruminococcus*, *Roseburia*, to be widely abundant. Among these, *Blautia*, *Butyrivibrio* and *Roseburia* have been associated with butyrate production and a positive contribution to the host [52]. The order *Clostridiales* within the class *Clostridia* is mainly responsible for short-chain fatty acid metabolism in the chicken caecum [53]. *Bacteroidia* were also common, including the genera *Bacteroides*, *Alistipes*, *Parabacteroides*, *Porphyromonas* and others, all of which contribute to propionate production in the caeca [52]. At order level, *Clostridiales* and *Bacteroidales* were both abundant (data not shown), although the proportionate representation of *Bacteroidales* was higher than reported in many previous studies [12, 14, 54], possibly as a consequence of high dietary maize inclusion. Deeper analysis found the genus *Bacteroides*, gram-negative obligatory anaerobic bacteria of the family *Bacteroidaceae*, were highly represented in most datasets in line with earlier studies [48, 55]. *Bacteroides* are generally associated with degradation of polysaccharides, especially starch and glucans [56, 57], and the formation of short-chain fatty acids [7]. The proportion of unclassified bacterial sequences was notably high in many of the samples, encouraging further studies focused on identification and determination of their role(s) or hazards in the enteric microbiota.

While geographic location was a major variable within principle coordinates analysis, separation by chicken breed or line also revealed distinct clusters indicating a host component in microbiome composition, in agreement with previous studies [58, 59]. Similarly, distinct LEfSe biomarkers also defined different chicken breeds or lines. The core caecal lumen microbiome was represented by 48 genera, including *Barnesiella*, *Butyricimonas*, *Pararevotella*, *Prevotella*, *Bacteroides*, *Clostridium*, *Ruminococcus*, *Alistipes*, *Eubacterium*, *Bacillus*, *Lactobacillus*, *Blautia* and *Cellulosilyticum*, all of which may contribute to chicken food conversion in terms of hydrolysing starch and other macromolecules, and the subsequent formation of short-chain fatty acids via fermentation which are absorbed by the host. All of these genera appeared as breed or line-specific LEfSe biomarkers at one or both locations, indicating a possible host genetic contribution. Potential pathogenic and/or zoonotic organisms within the genera *Helicobacter*, *Campylobacter*, *Ureaplasma*, *Eggerthella* and *Fusobacterium* were also detected with abundance > 0.1% in several chicken lines, although clinical disease was not reported [60]. LEfSe analysis further identified some of these genera as breed or line biomarkers, notably associating Kadaknath chickens raised at location 2 with the elevated occurrence of *Fusobacterium*, *Campylobacter*, *Cronobacter* and *Enterococcus*. The pathogenic bacteria detected could exert both direct and indirect influence on the host and its enteric microbiota. In addition to the risk posed as primary pathogens [61, 62], inflammatory immune responses induced by these pathogens will influence the intestinal environment and its bacterial communities. Further work will be required to determine the relative contributions from the host and these pathogenic bacteria. In contrast, genera associated with beneficial, sometime probiotic effects such as *Lactobacillus* [63] were associated as biomarkers with the commercial Cobb400 line. Using 0.1 and 1.0% sequence occurrence cut-off values, a small number of bacterial orders and genera were restricted at higher level occurrence to one or more chicken breeds. Such natural variation may also offer valuable phenotypes amenable to quantitative genetic analysis. Consideration of the indigenous breeds found 11 and 13 genera to represent frequencies in excess of 0.1% for Aseel and Kadaknath, respectively, one and five at a 1.0% cut-off. The occurrence of pathogenic bacteria is of particular relevance. Bacteria of the genus *Campylobacter* were detected above the 1.0% cut-off in Kadaknath (location 2) and Aseel (locations 1 and 2), but not in Cobb400 or Ross 308. Without undertaking a direct controlled *Campylobacter* challenge study, it is not possible to comment on the relative resistance or susceptibility of these breeds and lines, although there is some evidence for genetic control of *Campylobacter jejuni* colonisation in inbred chicken lines [64]. A larger sample size would permit an odds ratio assessment of occurrence and genetic analysis. Culture and detailed genetic characterisation of the *Campylobacter* species/strains circulating would also be valuable. A controlled study utilising a defined *C*. *jejuni* strain and sampling multiple locations within the enteric environment would be required for a definitive assessment of relative resistance or susceptibility.

The relative proportions of several bacteria were correlated in all chickens, regardless of genotype, and represented the core caecal microbiome. Potential pathogens such as those within the genus *Fusobacteria* were negatively correlated with genera associated with enhanced metabolism such as *Butyricicoccus*, *Pararevotella*, *Prevotella*, *Bacteroides*, *Clostridium*, *Ruminococcus*, *Alistipes*, *Eubacterium*, *Bacillus*, *Lactobacillus*, *Blautia*, *Cellulosilyticum* and *Pseudobutyrivibrio*, supporting the recognised role for commensal microbiota in chicken health. The characterisation of different bacterial genera as biomarkers associated with specific chicken breeds offers new phenotypes which may be interrogated by quantitative genetic analysis. For example, *Lactobacillus* and *Bacillus* showed positive correlation in the Cobb400, but were negatively correlated with most other genera in Kadaknath and presented a mixed response in the Aseel and Ross 308. Interestingly, the Kadaknath populations separated by location presented different LEfSe profiles. While location was clearly a significant variable, it is not possible to confirm whether the two Kadaknath groups represented a single homogenous population or two distinct sub-populations. Future chicken genotyping would be expected to resolve this question. Overall, the Kadaknath chickens consistently presented fewer OTUs and distinct PCoA clusters compared to the Aseel and commercial lines, indicating significant diversity.

## Conclusions

The study presented here provides an introduction to chicken breed-specific variation in enteric bacterial occurrence and diversity. The description of variation between global commercial lines and indigenous Indian chicken breeds offers a panel of phenotypes which may be amenable to genetic selection for use in breed improvement. Diversity within breeds may be of greatest interest where there is an opportunity to determine the genetic basis of varied bacterial occurrence. Improved understanding of host-microbiome interactions may support enhanced productivity from low value diets and greater resistance to colonisation by pathogenic and zoonotic organisms.

## Additional files

Additional file 1: Details of chicken lines (breed), location and farms sampled for evaluating the caecal microbiome. (XLSX 9 kb)

Additional file 2: Details of the primers used for amplicon preparation. (XLSX 9 kb)

Additional file 3: Sample wise details of the sequencing reads uploaded to MG-RAST for taxonomy assignment. TPC, TPA and TPK for Ross 300, Aseel and Kadaknath, respectively at Tamil Nadu location similarly APC, APA and APK for Cobb400, Aseel and Kadaknath, respectively at Anand location. (XLSX 13 kb)

Additional file 4: Rarefaction curves based on observed OTUs and Shannon index. Entire dataset of three primers were used and OTUs were clustered at > 97% identity using QIIME. A for OTUs and B for Shannon index. (TIF 1044 kb)

Additional file 5: Clustering analysis showing distinct clusters for two locations. Analysis performed using Bray-Curtis similarity method using PAST. Samples of all there primers were plotted. (TIF 6136 kb)

Additional file 6: Rarefaction curve based on observed OTUs for each primer pair. OTUs were clustered at > 97% identity using QIIME. (TIF 72 kb)

Additional file 7: Different alpha diversity indices for primer pair 1 (P1) (T4.1), 2 (P2) (T4.2) and 3 (P3) (T4.3) using QIIME where OTUs were clustered at 97% sequence similarity. (XLSX 18 kb)

Additional file 8: Representation of each phylum in the three primer pairs. Taxonomy classification of MG-RAST was used and abundance was calculated in terms of % mean relative frequency using STAMP. (TIF 42 kb)

Additional file 9: Taxon wise abundance of microorganisms for each primer pair in different breeds/lines. Taxonomy was assigned with RDP database, minimum *e* value of 1E-5 and identity of 80% using MG-RAST. (XLSX 10 kb)

Additional file 10: Pairwise comparison of *P* values by ANOSIM analysis based on Bray-Curtis Index using PAST. The *P* value was corrected with sequential Bonfferoni correction method. (XLSX 9 kb)

Additional file 11: Phylum (T7.1), class (T7.2) and genus (T7.3) level classification of caecal microbiome of chicken breeds/lines using Primer pair P2. Taxonomy was assigned using MG-RAST and subsequently analysed using STAMP where Benjamini-Hochberg test was used for multiple sample correction. Abundance was expressed in terms of % mean relative frequency. (XLSX 15 kb)

Additional file 12: Different alpha diversity indices for each sample of university farm data estimated using QIIME for primer pair P2 data. OTUs were clustered at > 97% similarity. (XLSX 12 kb)

Additional file 13: The core caecal microbiome of Indian chicken breeds. All sequences produced using primer pair P2 for each respective breed irrespective of location were pooled for this analysis. Genus abundance (% mean relative frequency) 0.1% (A) and 1.0% (B) were taken into account. List of all genera are given in Additional file 14. (TIF 371 kb)

Additional file 14: The core caecal microbiome of Indian chicken breeds. All sequences produced using primer pair P2 for each respective breed/line were used for analysis and the genus abundance (% mean relative frequency) 0.1% (A) and 1.0% (B) were taken into account. (XLSX 13 kb)

Additional file 15: Location specific biomarkers. (A) LEfSe analysis shows differentially abundant genera as biomarkers at two different locations determined using Kruskal-Wallis test (*P* < 0.05) with LDA score > 3.5. (B) Cladogram representation of the differentially abundant families and genera (only top 50% are plotted hare). The root of the cladogram denotes the domain bacteria. The taxonomic levels phylum and class are labelled, while family and genus are abbreviated, with the colours indicating the breed/line hosting the greatest abundance. The size of each node represents their relative abundance. (TIF 19652 kb)

Additional file 16: Correlation among the bacterial order detected in the caeca of different chicken breeds. Sequencing reads produced using primer pair P2 were pooled into a single pool for each breed, combining samples from different farm locations. A Pearson’s *r* correlation was expressed using METAGENassist. The breeds represented are A. Aseel, B. Cobb400, C. Ross 308 and D. Kadaknath. (TIF 1858 kb)
