# Supplementary figures and images for: Microbial diversity and community composition of caecal microbiota in commercial and indigenous Indian chickens determined using 16s rDNA amplicon sequencing
Source: Microbiome. 2018 Jun 23;6:115. doi: 10.1186/s40168-018-0501-9 (PMC6015460; doi:10.1186/s40168-018-0501-9)

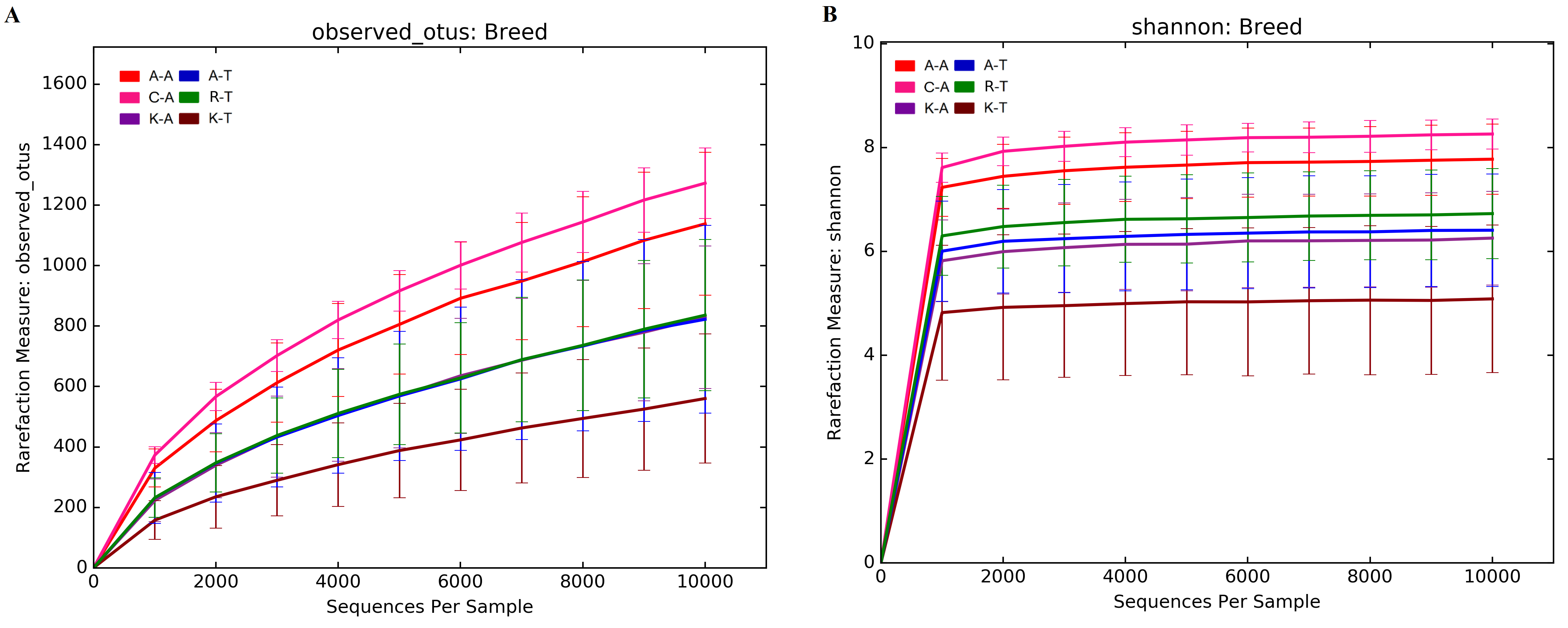

Supplement: Supplementary file 4 — Rarefaction curves based on observed OTUs and Shannon index. Entire dataset of three primers were used and OTUs were clustered at > 97% identity using QIIME. A for OTUs and B for Shannon index. (TIF 1044 kb) [file 40168_2018_501_MOESM4_ESM.tif]

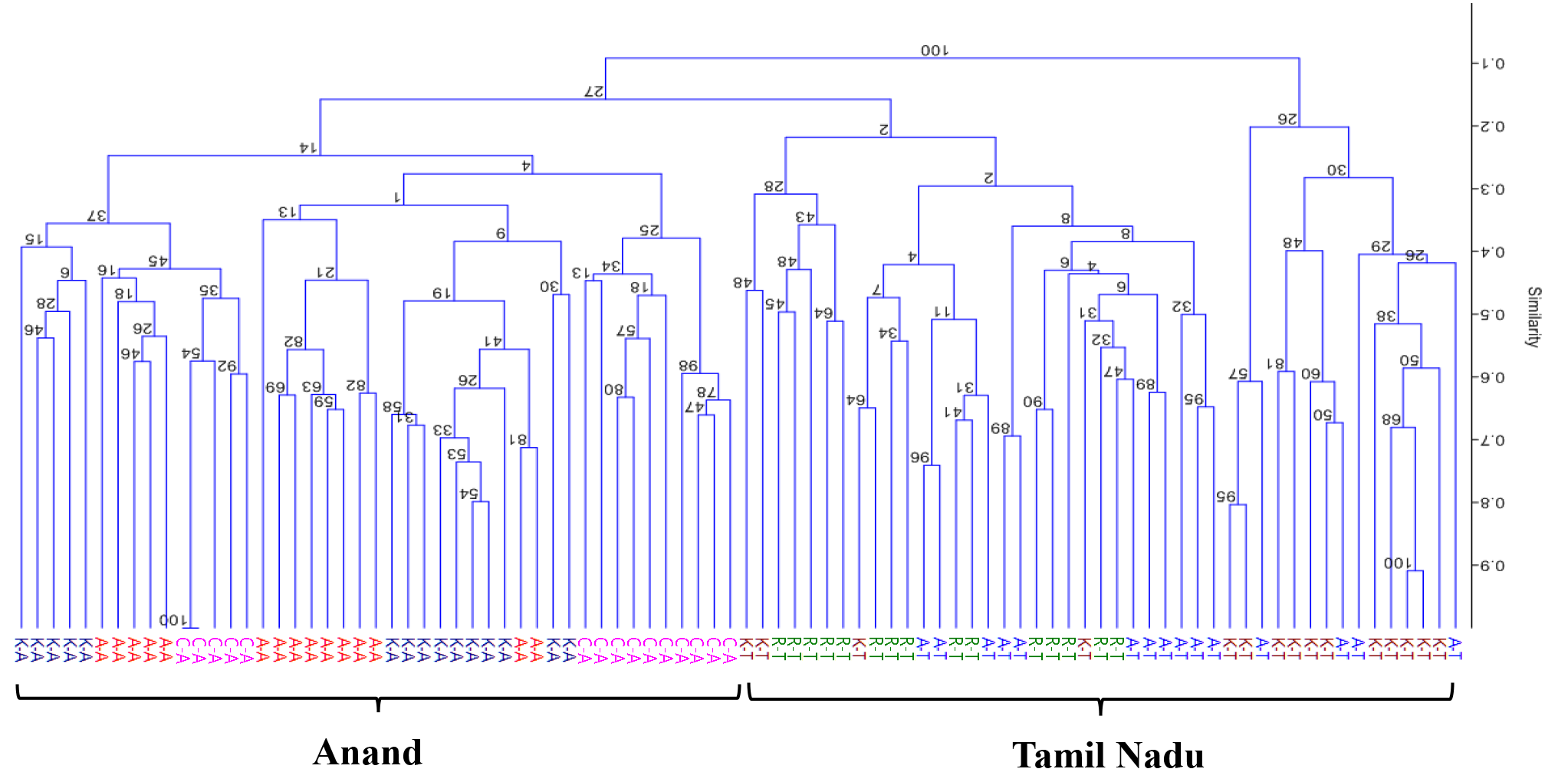

Supplement: Supplementary file 5 — Clustering analysis showing distinct clusters for two locations. Analysis performed using Bray-Curtis similarity method using PAST. Samples of all there primers were plotted. (TIF 6136 kb) [file 40168_2018_501_MOESM5_ESM.tif]

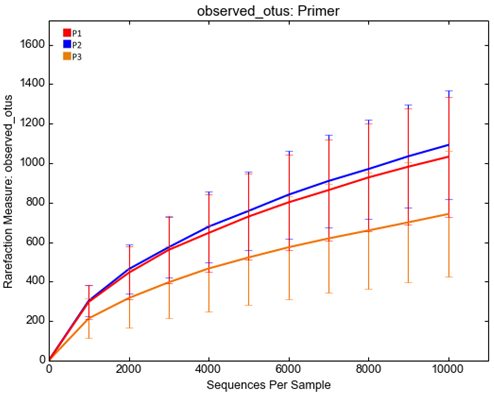

Supplement: Supplementary file 6 — Rarefaction curve based on observed OTUs for each primer pair. OTUs were clustered at > 97% identity using QIIME. (TIF 72 kb) [file 40168_2018_501_MOESM6_ESM.tif]

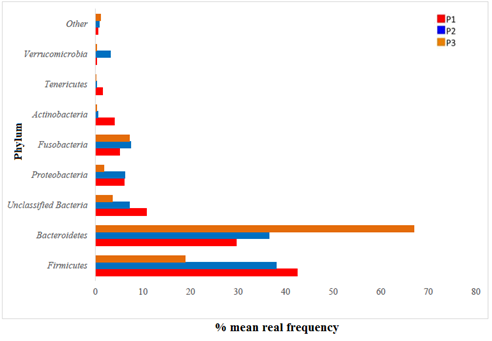

Supplement: Supplementary file 8 — Representation of each phylum in the three primer pairs. Taxonomy classification of MG-RAST was used and abundance was calculated in terms of % mean relative frequency using STAMP. (TIF 42 kb) [file 40168_2018_501_MOESM8_ESM.tif]

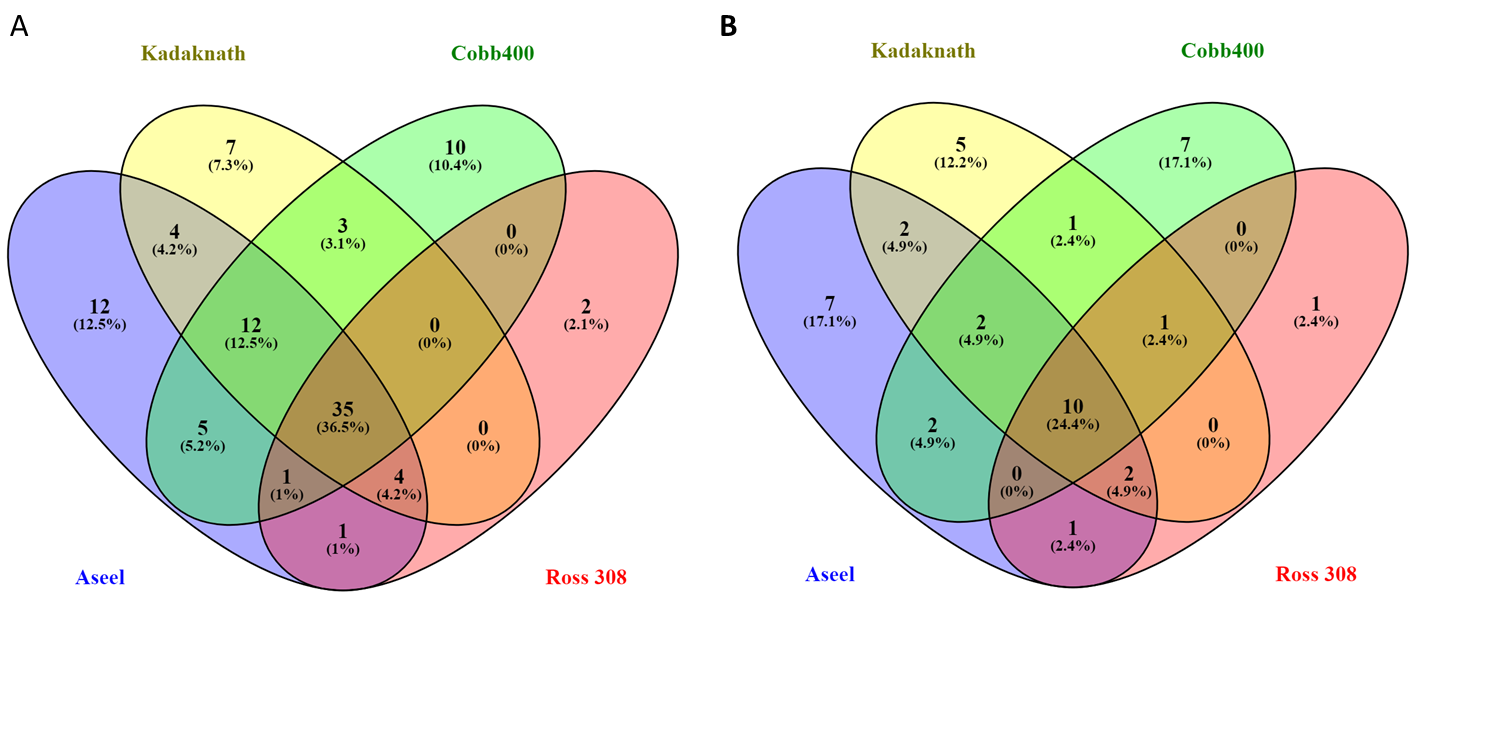

Supplement: Supplementary file 13 — The core caecal microbiome of Indian chicken breeds. All sequences produced using primer pair P2 for each respective breed irrespective of location were pooled for this analysis. Genus abundance (% mean relative frequency) 0.1% (A) and 1.0% (B) were taken into account. List of all genera are given in Additional file 14. (TIF 371 kb) [file 40168_2018_501_MOESM13_ESM.tif]

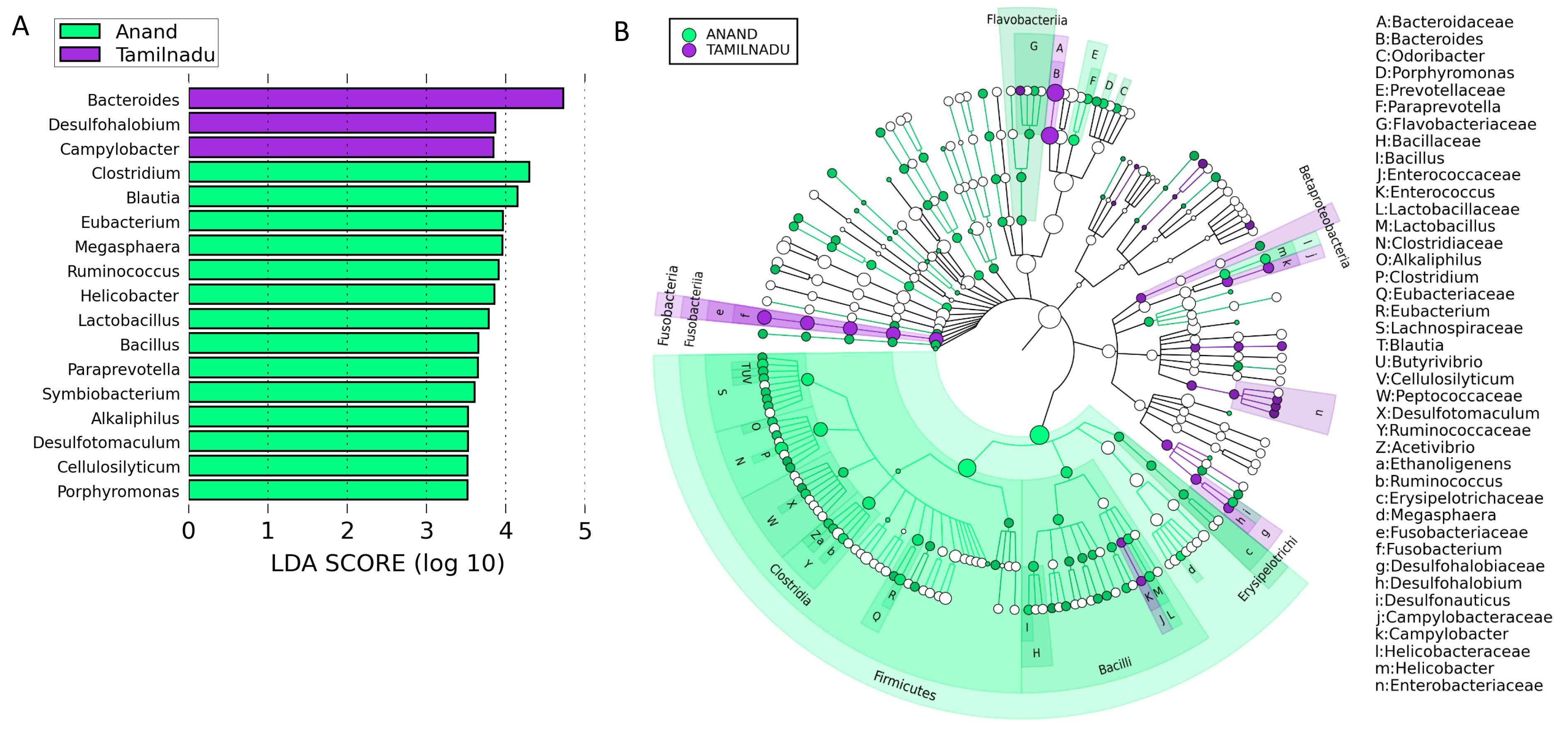

Supplement: Supplementary file 15 — Location specific biomarkers. (A) LEfSe analysis shows differentially abundant genera as biomarkers at two different locations determined using Kruskal-Wallis test (P < 0.05) with LDA score > 3.5. (B) Cladogram representation of the differentially abundant families and genera (only top 50% are plotted hare). The root of the cladogram denotes the domain bacteria. The taxonomic levels phylum and class are labelled, while family and genus are abbreviated, with the colours indicating the breed/line hosting the greatest abundance. The size of each node represents their relative abundance. (TIF 19652 kb) [file 40168_2018_501_MOESM15_ESM.tif]

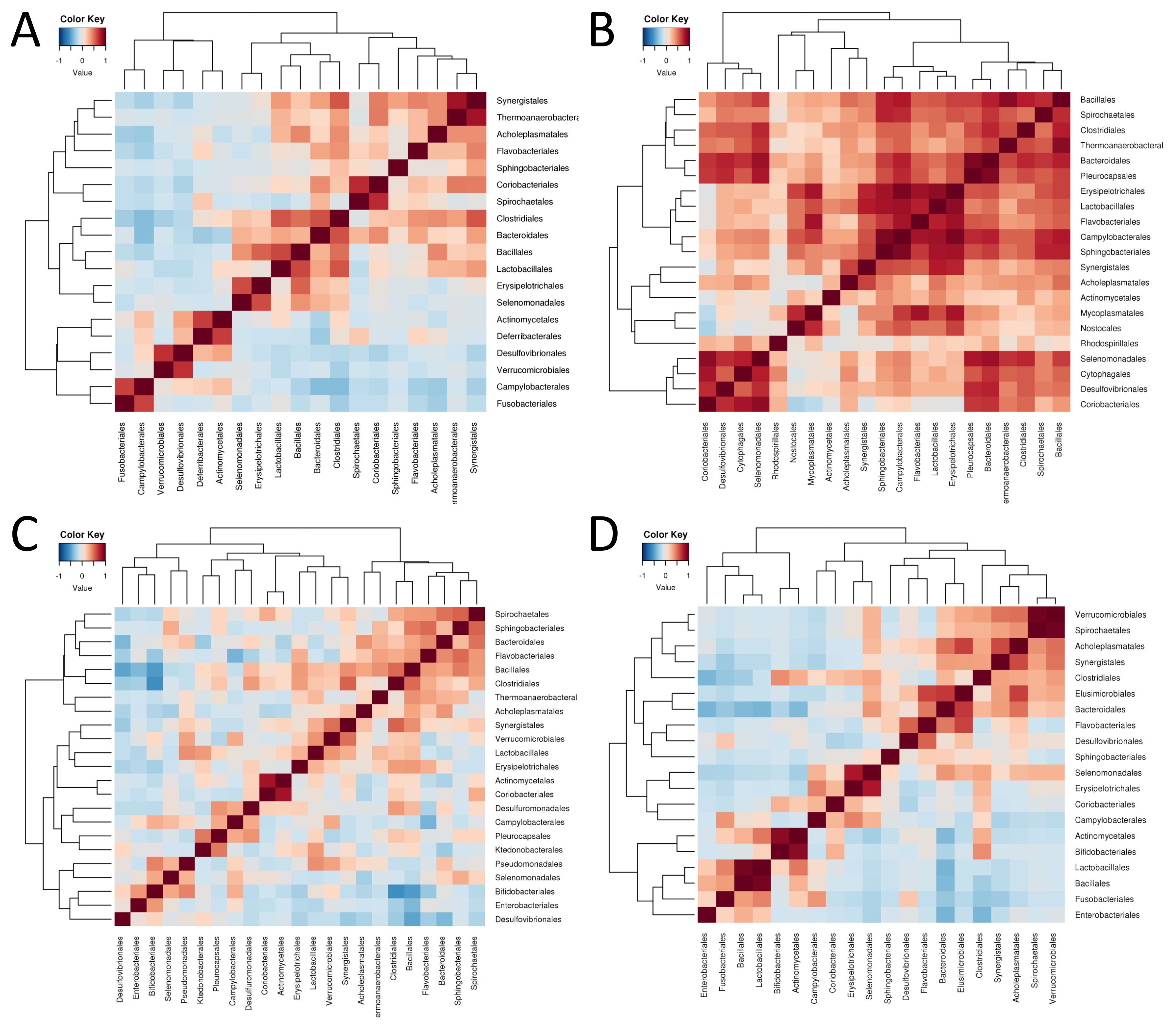

Supplement: Supplementary file 16 — Correlation among the bacterial order detected in the caeca of different chicken breeds. Sequencing reads produced using primer pair P2 were pooled into a single pool for each breed, combining samples from different farm locations. A Pearson’s r correlation was expressed using METAGENassist. The breeds represented are A. Aseel, B. Cobb400, C. Ross 308 and D. Kadaknath. (TIF 1858 kb) [file 40168_2018_501_MOESM16_ESM.tif]
